# Videographic Assessment of Glaucoma Drop Instillation

**DOI:** 10.5005/jp-journals-10008-1183

**Published:** 2015-09-25

**Authors:** Gabriel Lazcano-Gomez, Armando Castillejos, Malik Kahook, Jesus Jimenez-Roman, Roberto Gonzalez-Salinas

**Affiliations:** Professor, Department of Ophthalmology, Glaucoma Service, Association to Prevent Blindness in Mexico, Mexico City, Mexico; Professor, Department of Ophthalmology, Glaucoma Service, Association to Prevent Blindness in Mexico, Mexico City, Mexico; Professor, Vice Chair and Director, Department of Ophthalmology, School of Medicine, University of Colorado, Anschutz Medical Campus, Aurora, USA; Professor, Department of Ophthalmology, Glaucoma Service, Association to Prevent Blindness in Mexico, Mexico City, Mexico; Associate Researcher, Department of Biomedical Research, Faculty of Medicine Autonomous University of Queretaro, Queretaro, Mexico

**Keywords:** Education, Instillation, Intervention, Short-term, Technique.

## Abstract

**Purpose:** To assess the effect of patient education on videotaped topical instillation of artificial tear drops on subsequent topical instillation.

**Materials and methods:** Forty-five patients, who had been using glaucoma drops for at least 6 months and with a best-corrected visual acuity of 20/100 or better, were studied. The patients were asked to instill an artificial tear drop using their accustomed technique while being video recorded. The patients viewed the recordings, and the errors in their drop instillation method were pointed out. This was followed by an educational session on proper drop instillation technique. After 30 minutes, patients were videotaped instilling drops to ascertain the effect of the educational session. The variables compared were: number of drops instilled, number of drops reaching the ocular surface, and the number of times the tip of the medication bottle touched the eye or ocular adnexa.

**Results:** Before the instruction session, patients squeezed an average of 1.5 ± 0.9 drops from the bottle, and the average number of drops reaching the conjunctival fornix was 0.9 ± 0.7. The tip of the bottle touched the ocular adnexa in 29/45 (64.4%) patients. After the education session, the patients squeezed an average of 1.2 ± 0.5 drops and an average of 1.2 ± 0.4 drops reached the conjunctival fornix. The tip of the bottle touched the ocular adnexa in 13/45 (28.9%) patients. With proper instructions, the percentage of patients that instilled just one drop on the eye increased from 66 to 82%.

**Conclusion:** A single educational session on the proper use of topical drops improves the successful instillation of eye drops. However, it was not determined whether the patients will retain the improved instillation technique for long-term or if the intervention results in only a short-term improvement.

**How to cite this article:** Lazcano-Gomez G, Castillejos A, Kahook M, Jimenez-Roman J, Gonzalez-Salinas R. Video-graphic Assessment of Glaucoma Drop Instillation. J Curr Glaucoma Pract 2015;9(2):47-50.

## INTRODUCTION

Lowering intraocular pressure (IOP) in patients with glaucoma or ocular hypertension is known to slow the development and progression of the glaucomatous disease process.^1,2^ However, it is well known that poor adherence to the prescribed therapy can result in failure to slow disease progression.^3^ Poor adherence to prescribed therapeutic regimens is a common problem in medical therapy and is reported in up to 70 to 75% of patients with chronic diseases.^4-6^ Glaucoma patients are generally older and may have physical and visual limitations, which can worsen the problem.^7,8^

The risk factors associated with poor adherence are many and poorly understood, although many recent studies have tried to identify specific causes.^9-11^ Even though important progress has been made in this field, most studies arrive at similar conclusions, *viz,* adherence is generally poor and quite common. The causes for non-adherence are many―advanced age, concomitant disease, socioeconomic status, cost of the therapy and complexity of the dosing regimen; only worsening the problem.

An important part of poor adherence is an incorrect dosing technique. The inherent difficulties of delivering medications using multidose eyedrop bottles have been reviewed in two studies.^12,13^ In one study, only 39% of patients with glaucoma administered the drops successfully, i.e. instilling just one drop on the ocular surface without touching the ocular adnexa.^14^ Poor manual dexterity and reduced vision worsened the problem, which is why one study reported that 17% of patients relied on another person to administer the drops.^12^

Patient education can play an important role in achieving a proper eyedrop dosing technique. In addition to education, regular monitoring of how the patient applies the drops might ensure that the information has been retained.

Thus, the purpose of this study was to determine the effectiveness of a training session on the subsequent instillation of topical medications. To accomplish this, we videorecorded the dosing method used by glaucoma patients who were chronically using topical IOP lowering therapy. After the initial video documentation, the patient viewed the recordings with the instructor who pointed out the mistakes. The correct proper eyedrop method was then demonstrated, and the patient was videotaped again 30 minutes later to evaluate the effectiveness of the training session.

## MATERIALS AND METHODS

The protocol was approved by the internal review board of the ‘Asociacion para Evitar la Ceguera en Mexico IAP’, research funds were arranged and the study conducted in the glaucoma department of the same institution. The procedures conformed to the tenants of the declaration of Helsinki, and a signed informed consent form was obtained from all the participants.

We included patients with a diagnosis of primary open-angle glaucoma (POAG), primary angle-closure glaucoma (PACG) or ocular hypertension (OHT), who had been using glaucoma eyedrops for at least 6 months. All patients had a best-corrected visual acuity (BCVA) of 20/100 or better in both eyes. The exclusion criteria included secondary causes of glaucoma and the presence of systemic diseases which might influence drop instillation technique, such as arthritis or tremors. Age, sex and diagnosis were recorded at the time of enrollment.

All the patients were asked to instill an artificial tear drop (sodium hyaluronate) using the same technique they used at home. The application of the drop was videotaped, and one observer recorded all details of the instillation technique, including―the number of drops squeezed out from the bottle, the number of drops that fell directly onto the conjunctival fornix or cornea, and the number of times the tip of the bottle touched eyelashes, skin or conjunctiva.

### Educational Session

After the initial drop instillation, patients were instructed on the correct technique for eyedrop instillation. Each of the patients received instillation instructions by the same ophthalmologist (Lazcano-Gomez Gabriel) in an examining room of the glaucoma service. The correct technique for all patients was holding the lower lid down with one or two fingers while instilling one drop on the conjunctival fornix or corneal surface, without the bottle tip touching the ocular surface or periocular tissues. The educational session was not videotaped. The time required to get the patient to achieve the correct technique, depended on the ability of each patient to apply the drop.

After 30 minutes, we asked the patient to instill a drop of the same artificial tear to ascertain the effects of the teaching session on each patient. The second instillation was videotaped for comparison with the first attempt. The number of drops squeezed out from the bottle, the number of drops that fell directly onto the conjunctival fornix or cornea, and the times the tip of the bottle touched eyelashes, skin or conjunctiva were recorded. The patients were allowed to sit, stand, or use their preferred position during the application of the eyedrops.

A correct instillation of the eyedrop was defined as the instillation of one drop into the conjunctival fornix without the tip of the bottle touching any tissues. All data was tabulated using Microsoft Excel 97-2000.

### Statistical Analyses

Statistical analyses were performed with the statistical package for social sciences (SPSS) software (version 20, SPSS, Inc, Chicago, IL, USA). Continuous variables are presented as the ‘means ± standard deviations (SDs)’ and the categorical variables as percentages. Student’s t-tests were used to determine the significance of the differences between means in the variables that were normally distributed. The Wilcoxon signed-rank tests were used for paired samples when the data were not normally distributed. Nominal dichotomous variables were assessed by McNemar tests. The p-values < 0.05 were considered to be statistically significant. Normal and non-normal distributions were determined by Kolgomorov-Smirnov tests for all variables.

## RESULTS

Forty-five patients were studied of which 38 were women (84.4%). Their mean age was 56.8 ± 15.1 years with a range from 25 to 86 years. Twenty-nine patients (64.4%) had POAG, nine (20%) had PACG, and seven (15.6%) had OHT. All the patients had uncorrected visual acuity better than 20/100, and had at least 6 months of self-administration of glaucoma eyedrops. None of the patients had physical limitations due to systemic diseases that could influence the drop instillation.

Before the education session, the mean number of drops instilled/patient was 1.5 ± 0.9 with a range of 1 to 4 drops. Thirty patients (66.7%) correctly instilled one drop as instructed. The mean number of drops that were correctly instilled directly on cornea or inferior conjunctival fornix/patient was 0.9 ± 0.7 with a range of 0 to 4 drops. In 29 patients (64.4%), the tip of the bottle touched eyelashes, conjunctiva, or other ocular tissue for 1.7 ± 2.2 times/patient with a range of 0 to 8 times.

Thirty minutes after the educational session, the mean number of drops instilled/patient decreased significantly to 1.2 ± 0.5 with a range of 1 to 3 drops (p = 0.011), with 37 patients (82.2%) instilling one drop as instructed. The mean number of drops instilled directly on the cornea or inferior conjunctival sac/patient changed from 0.9 ± 0.7 to 1.2 ± 0.4 with a range of 1 to 3 drops (p = 0.063). Only 13 patients (28.9%) were noted to touch the bottle tip to surrounding tissues as compared to 29 (64.4%) patients prior to the educational session (p = 0.05), with a frequency of 0.5 ± 1.0 times/patient and a range of 0 to 4 times (p = 0.0001).

## DISCUSSION

Our results show that an organized educational session involving ‘viewing videotaped topical instillation’ of artificial tears in experienced patients with glaucoma resulted in better technique. The improvement was in avoiding the application of excessive number of drops on the eye, while decreasing the chances of touching the tip of the bottle to the surrounding tissues. This improvement can have important implications for disease management and safety of using topical drops in patients who are chronically using topical eyedrops to manage their ophthalmic diseases. It is possible that the lack of response to treatment in some patients might be due to failure of the drug to reach the targeted tissue.

Poor adherence to therapy in general can be categorized as either intentional or unintentional.^13-15^ The reasons for intentional non-adherence can include: refusal to take medications because of a feeling of having recovered from the illness (denial) or a failure to fill prescriptions given by the treating physician.^15^ The unintentional reasons for non-adherence to therapy might include: lack of money to purchase medications, physical limitations, forgetfulness, and improper instillation techniques. While many of the reasons for non-adherence are difficult to modify, the educational sessions for improving instillation techniques are easy and cost-effective intervention that can potentially rectify a major reason for poor outcomes from the prescribed medications.

Our results showed that the educational instillation technique was effective in 82% of the patients instilling only one drop as comparison to 66% in the pre-educational evaluation. This agrees with the study of Liu et al who examined the effect of education on the ability of post-cataract patients to administer eyedrops correctly. Of the 133 patients who received an educational session on eyedrop instillation, 112 (84%) were more proficient on postoperative day 30 as compared to 29/49 patients (59%) who had not received the education.^16^

Brown et al studied the self-administration of topical medications in glaucoma patients and found that 21% of patients administered two or more drops when only intending to use one drop.^17^ Kass et al reported a mean of 2.4 drops being dispensed per eye per treatment, and a mean of 1.98 drops reaching the conjunctival fornix per eye per treatment.^18^ However, in our study, the number of patients that instilled only one drop increased to 82% with an average of 1.22 drops per application and 1.15 drops falling directly on the conjunctival fornix. These findings were significantly different from those prior to the education session.

The risks associated with the dropper tip touching the eye or ocular adnexa cannot be overstated. The touching of the tip can be associated with corneal abrasions and infectious keratoconjunctivitis, associated with bottle contamination.^19^

Many other authors have studied the eyedrop application technique in patients with eye diseases. Hennessy et al evaluated 204 patients with moderate to severe glaucoma visual field damage and reported that 80% of the patients stated that they had no difficulty instilling eye drops, but only 39% of patients did instill only one drop onto the ocular surface without the bottle tip touching the adnexa.^9^ In their 2011 study, Hennessy et al evaluated self-administration of both visually impaired glaucoma and retina patients. Approximately 30% of patients from both groups could not get a drop onto the eye, with a success rate of 39% in glaucoma group *vs* 31°% in retina group.^20^ Sleath et al studied the relationship between medication adherence and eyedrop technique using a microelectromechanical systems (MEMS) device and visual field alterations, and concluded that only 38% of 102 patients had a perfect drop technique. They reported that non-white patients were more likely to be less than 80% adherent to their treatment. This resulted in greater visual field damage in these patients.^21^

Some other factors, like―poor information about glaucoma, consequences of not using the medications properly, and the adverse effects of the medication can result in low adherence to glaucoma treatment as demonstrated by Friedman et al. Other factors affecting the compliance of patients were cost of the medications, receiving samples and ethnicity.^22^

Our study has limitations. Firstly, we did not classify the patients by age group or severity of glaucoma damage. Some patients might have had difficulty with drops instillation associated with specific visual field defects. In addition, we did not investigate the socioeconomic and educational level of the patients, a factor that could determine level of understanding and correct application of the drugs. Furthermore, the fact that patients were evaluated by video recordings probably induced instillation technique modifications related to stress. Another important limitation of this study was the short period between the two evaluations of the instillation technique. Thus, the long-term educational effects could not be evaluated. These issues can be addressed and better determined in future studies.

## CONCLUSION

Proper administration of topical glaucoma medication delivers better therapeutic results. Instructing patients into the appropiate drop instillation technique, in addition to an active monitoring of patients in a clinical setting, are simple interventions which do enhance therapeutic outcomes. We suggest that future studies focus on how specific populations of patients respond to the same educational intervention, and whether the patients exhibit a lasting effect on their drop instillation habits after the education has been given.
